# A 15-year retrospective review of urodynamic studies in children at Red Cross War Memorial Children’s Hospital, Cape town, South Africa

**DOI:** 10.1186/s12887-022-03462-4

**Published:** 2022-07-08

**Authors:** Thembisile Dintle Mosalakatane, Mignon McCulloch, Peter Nourse, Ashton Coetzee, Anne Wright, Jeanette Raad, John Lazarus, Justin Howlett

**Affiliations:** 1 Division of Paediatric Nephrology, School of Child and Adolescent Health, Red Cross War Memorial Children’s Hospital, University of Cape Town, Western Cape, Cape Town, 7700 South Africa; 2 Children’s Bladder Clinic, Evelina London Children’s Hospital (Guy’s and St Thomas’ NHS Foundation Trust), London, England; 3 Urodynamic and Manometric Unit, Red Cross War Memorial Children’s Hospital, University of Cape Town, Western Cape, Cape Town, 7700 South Africa; 4 Division of Urology, Red Cross War Memorial Children’s Hospital, University of Cape Town, Western Cape, Cape Town, 7700 South Africa

**Keywords:** Urodynamic Study, LUTD, ICCS, DSD, NDO, DLPP, NLUTD

## Abstract

**Background:**

Despite the undeniable diagnostic benefits of urodynamic studies (UDS), their adoption into clinical practice in Africa has been slow. This study aimed to review the use of invasive UDS in children at a tertiary paediatric hospital in South Africa.

**Methods:**

A retrospective analysis of 1108 UDS was conducted. Patient demographic characteristics, primary diagnosis, indication and urodynamic outcomes were reviewed. Presence of urodynamic high-risk features were documented, and a comparison was made between the first study and follow-up study.

**Results:**

This study revealed increasing trends in the use of UDS from 2015. Referrals were from Urology (37.7%), Spinal defects clinic (34.4%), Nephrology (20.8%) and other departments (7.0%). The most common reason for referral was review of medical treatment (36.5%). Spinal dysraphism (58.3%) accounted for the majority of conditions seen. Majority (59.1%) of the patients were receiving more than one type of bladder treatment at the time of their first study, with clean intermittent catheterisation (46.5%) being the most common form of bladder management. 97.5% of studies were performed using transurethral bladder catheterization. Urodynamic diagnosis was neurogenic in 74.0%, anatomical (12.2%), functional (8.8%) and normal (5.0%). There was statistically significant improvement in bladder compliance, detrusor leak point pressure and detrusor sphincter dyssynergia between the first study and a subsequent study following therapeutic intervention.

**Conclusions:**

The unique ability of UDS to demonstrate changes in detrusor pressures, which is a common reason for therapy failure, makes UDS an invaluable tool in the diagnosis and management of children with lower urinary tract dysfunction.

**Supplementary Information:**

The online version contains supplementary material available at 10.1186/s12887-022-03462-4.

## Background

Lower urinary tract dysfunction (LUTD) is a common problem causing a major social and psychological burden to both children and their families. If left untreated, some cases of LUTD such as anatomic, neurogenic or severe dysfunctional voiding, may cause irreversible kidney damage. The goal of treatment is therefore aimed at protecting the kidneys and ensuring urinary continence, with direct positive effects for the child’s quality of life [1]. The treatment of LUTD greatly depends upon establishing the correct diagnosis. Traditionally, the evaluation of a child with LUTD includes a history, physical examination, bladder and stool diaries, kidney-ureter-bladder (KUB) ultrasonography and voiding cystourethrogram (VCUG). Unfortunately, there is a sizable proportion of patients in whom conventional approaches fail to provide an explanation of their symptoms.

Urodynamic study (UDS) is used to identify other lower urinary tract (LUT) pathologies where conventional modalities have failed to establish a diagnosis. The major benefit of UDS is its ability to assess the mechanical function of the bladder, sphincter and urethra [2]. Invasive UDS has an advantage over other modalities as the only investigation that is able to assess detrusor pressures during bladder filling and voiding phases. When VUDS is performed, information on both mechanical function and anatomy of the LUT can be obtained [2, 3]. There is compelling data that support its use in patients with neurogenic lower urinary tract dysfunction (NLUTD) [4, 5]. The International Children’s Continence Society (ICCS) now recommends UDS (preferentially VUDS) for all children with spinal dysraphism and those with suspected neurogenic bladder from other causes [4]. Several institutions have now begun to adopt universal rather than risk stratified UDS protocols for children with spinal dysraphism [6].

Invasive UDS is not without its own inherent problems. It involves bladder catheterization, which may cause discomfort and anxiety in a child. Many authors do not recommend its routine use in assessing children with non-neurogenic lower urinary tract dysfunction (NNLUTD) [7, 8]. They argue that UDS does not generally change the management and treatment in these patients, as in most cases a detailed voiding history and physical examination is usually sufficient for a correct diagnosis [7, 8]. It is therefore, suggested that, in these types of patients, UDS be reserved for children who are failing standard therapy or where conventional investigations have failed to provide answers for their symptoms [9]. Some authors support its use in the evaluation of children with recurrent urinary tract infections (UTI) associated with history of voiding dysfunction (frequency, urgency and incontinence) [10, 11].

As the role and demand of UDS in both paediatric urology and nephrology increases, several studies have exposed gaps in the literature [2, 12–14]. There is also lack of data in the use of UDS in the African context, and current knowledge on urodynamic investigations in the African paediatric population is mainly based on studies done in Europe, North America and Asia. This study was undertaken to describe the 15-year experience with the use of invasive urodynamic studies conducted at Red Cross War Memorial Children’s Hospital (RCWMCH), Cape Town, South Africa during the period September 2005 to September 2020.

The specific objectives for this study are:

1. To identify the common indications for urodynamic studies at RCWMCH.
2. To determine the prevalence and aetiology of both non-neurogenic and neurogenic lower urinary tract dysfunction in children undergoing urodynamics investigation at RCWMCH.
3. To determine the proportion of patients with active bladder interventions such as clean intermittent catheterization (CIC), antimuscarinic/ anticholinergic medication, intravesical Botox injection, Deflux®(Hyaluronic acid/Dextranome)/STING (subureteral Teflon injection) procedure, ureteral reimplantation and bladder augmentation.
4. To determine the proportion of patients with high-risk features [low bladder compliance, detrusor leak point pressure (DLPP) ≥ 30 cm H_2_O, presence of neurogenic detrusor overactivity (NDO), presence of detrusor sphincter dyssynergia (DSD)] for upper urinary tract damage.

## Methods

### Study site

The RCWMCH Urodynamics & Manometric Unit was established in September 2005. Initially the unit provided services mainly to the Spinal Defect Clinic. The unit has expanded over the years to include investigation of children with LUTD due to causes other than spinal dysraphism. Services offered include Uroflowmetry, Urodynamics, Video Urodynamics, Ambulatory Urodynamics, pH Impedance Studies, and Anorectal Manometry. Urodynamic testing is performed by a trained medical technologist, who has undergone training in urodynamics. Until the year 2016, the studies were performed using the Medtronic Urodynamic Measurement System. The urodynamic studies are now performed using the Nexam Pro Urodynamic System. It is the standard practice of the hospital to perform UDS following the ICCS good urodynamic practices [12]. All studies are performed and interpreted by a urodynamic technologist and reviewed by an experienced urologist. Complex cases or inconclusive studies are usually discussed during weekly combined (radiology, urology and nephrology) meetings.

### Study population

This work covers a fifteen-year period from September 2005 to September 2020. Included in this study are all patients who underwent invasive UDS at RCWMCH during the study period. Patients were excluded from the analysis if studies were partially completed or in cases where data was missing from their records.

### Data collection

From the request form completed by the referring clinician the following demographic and clinical information was retrieved: 1) patient demographic data at the time of UDS (age, sex,); 2) referring speciality; 3) the type of study (first study or follow-up); 4) primary diagnosis; 5) reason for referral; and 6) patient’s current treatment (CIC, pharmacological, surgical). Missing data was accessed through the patient’s hospital record. The UDS report for each patient was then reviewed and the following data was recorded. 1) Maximum cystometric capacity ( expressed as % of expected bladder capacity); 2) presence of high risk features (low bladder compliance, DLPP ≥ 30 cm H_2_O, NDO, DSD); and 3) VUR. The UDS findings were classified into normal, neurogenic, functional and anatomical. Study data was collected and managed using REDCap electronic data capture tools hosted at University of Cape Town (redcap@uct.ac.za).

### Definitions

‘‘Terminology adheres to standards recommended by the ICCS except where specifically noted [13].’’

### End-fill detrusor pressure (EFP*)*

The baseline detrusor pressure recorded at the end of the filling phase, prior to commencement of voiding.

### Low bladder compliance

Bladder compliance describes the relationship between changes in bladder volume and changes in detrusor pressure (mL/cmH_2_O) [13]. There are no standardised normal values for calculated compliance in children. This is because normal bladder capacity increases with age. In this study low bladder compliance was defined as EFP greater than 20 cm H_2_O (baseline detrusor pressure at the end of cystometry filling in the absence of detrusor overactivity) as a cut-off point. This is lower than the most frequently quoted risk level of 40 cm H_2_O. Of recent, many clinicians are revising this cut-off value and considering lower cut-off values to facilitate the chance of reversibility with treatment.

### Expected bladder capacity (EBC*)*

EBC was calculated from the Hjalmas equation [*EBC* = *age (years)* × *30* + *30 (expressed in ml*)] [12].

Definition of vesicoureteral reflux (VUR) is according to the International System of Radiologic Grading of VUR [15]:

I. High grade VUR: Refers to grade IV and V VUR
II. Low grade VUR: Refers to grade I, II and III VUR

### Data analysis

The analysis was performed using IBM Statistical Package for Social Sciences (SPSS) Statistics for Windows, Version 27.0. Continuous variables were expressed as range and median and the categorical variable as proportions n (%). *p* values were calculated by, χ. test, or Fisher’s exact test, as appropriate. *p*-value of 0.05 was considered statistically significant.

### Ethical approval

Ethical approval for this study was obtained from Human Research Ethics Committee, University of Cape Town *(HREC REF: 461/2020)* and Research Review Committee, Red Cross War Memorial Children’s Hospital *(RXH: RCC 239).*

## Results

### Study population and demographic characteristics

**Fig. 1:**
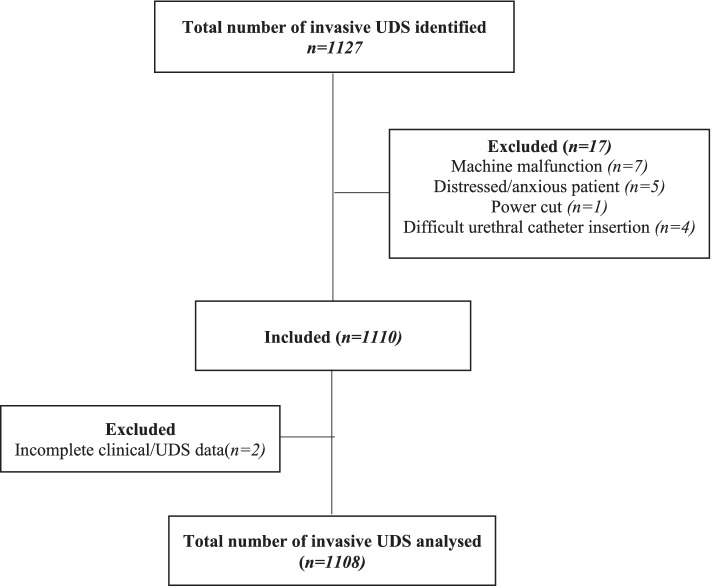
Study flow diagram outlining the sequence of records selection for the study

During the period under review, 1127 invasive urodynamic studies performed at RCWMCH were identified. As shown in Fig. 1,

exclusions were predominantly incomplete studies due to machine malfunctioning (7/1127), power cuts (1/1127), difficult urethral catheterisation (4/1127) and an uncooperative child (5/1127). Only 2/1127 cases were excluded for lack of complete clinical and/or UDS data. This number may not be a true reflection of the total number of incomplete studies as unsuccessful studies conducted during the initial setup phase of the urodynamic unit were not recorded. Primary analyses were performed using 1108 UDS studies: 646 (58.3%) male patients and 462 (41.7%) female patients. They had a median age of 7.0 years (IQR) at time of study (see Table 1).

**Table 1:** Demographic and clinical information in children referred for urodynamic studies

*Variable*	*n*	%
**Sex**		
Male	646	58.3
Female	462	41.7
**Age (years)**		
Median (IQR)	7.0(4.0–11.0)	
**Type of study**		
First study	727	65.6
Follow-up study	381	34.4
**Referral**		
Spinal Defect Clinic	381	34.4
Urology	418	37.7
Nephrology	231	20.8
Other departments	78	7.0
**Primary Diagnosis**^**a**^		
Spinal dysraphism	646	58.3
Acquired spinal abnormalities	52	4.7
Anorectal Malformation	54	4.9
Sacral agenesis	57	5.1
PUV	153	13.8
Primary VUR	17	1.5
Enuresis	49	4.4
Other	144	13.0
**Indication for UDS**^**b**^		
Baseline	116	10.5
Recurrent UTI	206	18.6
VUR	124	11.2
Recurrent UTI + VUR	109	9.9
Review medical therapy	404	36.5
Review surgical treatment	94	8.5
Pre-surgical intervention	63	5.7
Pre- transplant	43	3.9
Post-transplant	34	3.1
Other	51	4.6

^a^Some children had multiple conditions

^b^Some children had multiple indications

*IQR* Interquartile range

### Trend in the number of invasive urodynamics studies performed

**Fig. 2:**
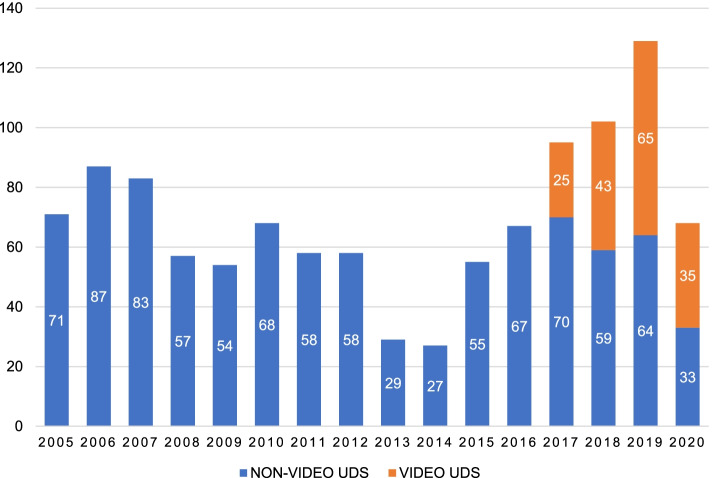
The number of invasive urodynamic studies performed at Red Cross War Memorial Children’s Hospital by year

There is variation in the number of studies performed annually (see Fig. 2). This variation in UDS quantity has been influenced by several factors including delays and interruptions caused by servicing of machine in 2009, unit renovations between 2013 and 2014, and more recently the disruption caused by the Covid-19 pandemic in 2020. Despite this variation in quantity of UDS performed per year, on average the number of studies increased from 2015 through 2019.

### General characteristics of children

Table 1 also summaries the clinical characteristics and indication for UDS. The most frequent conditions seen included spinal dysraphism (myelomeningocele, myelocele, lipomyelocele, disatematomyelia) 646 (58.3%), PUV 153(13.8), sacral agenesis 57(5.1%), anorectal malformation 54 (4.9%), acquired spinal abnormalities (trauma, infections etc.) 52 (4.7%) enuresis 49 (4.4%), primary VUR (1.5%), and other conditions (13.0%). More than one condition per patient could be present, for instance, a patient with spinal dysraphism could also have anorectal malformation.

Referrals were received from various departments, the majority coming from the Urology department (37.7%). The other sources of referral were Spinal defect clinic (34.4%), Nephrology (20.8%) and other departments (neurology, neurosurgery, oncology, other hospitals) (7.0%). The most common reason stated in the referral was review of medical treatment (36.5%).

### Types of bladder intervention at first study

**Fig. 3:**
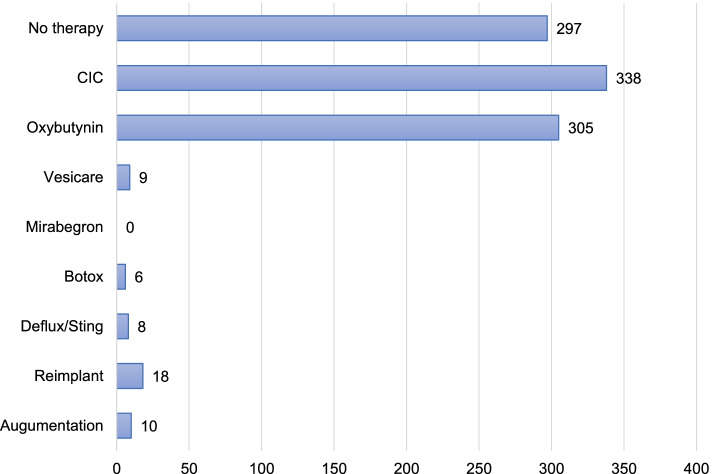
The various types of bladder management at first study (*n*=727)

As shown in Fig. 3, 297 (40.9%) of the patients were not on any therapy at the time of their first UDS. Of the 3 types of treatment (CIC, medical and surgical), CIC was found to be the most common form of bladder management 338 (46.5%). The majority of the patients 430 (59.1%) were receiving more than one type of bladder treatment.

### Method of bladder catheterisation

Almost all (97.5%) of the patients had bladder catheters inserted urethrally. Other methods of bladder catheterization were used if the patient was already using the technique for bladder emptying.

### Urodynamic outcomes

The urodynamic diagnosis was neurogenic in 820 (74.0%), anatomical 135 (12.2%), functional 98 (8.8%) and normal 55 (5.0%). Table 2 shows the distribution of urodynamic data of the 4 groups. There was no significant age difference in all the 4 groups. Although the majority of the studies were performed in males, there was a female (69.4%) predominance in those with functional LUTD. Almost all those with anatomical LUTD were males (97.0%). UDS revealed low bladder compliance in 44.4% of all studies. Detrusor overactivity was recorded in 14.4% of UDS. VUR was detected in 54 out of 168 (32%) VUDS.

**Table 2:** Urodynamic outcomes

*Variable*	*Total (%) n* = *1108*	*Normal (%) n* = *55*	*Neurogenic (%) n* = *820*	*Functional (%) n* = *98*	*Anatomical (%) n* = *135*
**Age (years)**					
Median (IQR)	7.0(4.0–11.0)	6.0(1.0–10.0)	7.0(4.0–11.0)	8.0(6.0–11.0)	8.0(5–11.0)
**Sex**					
Male	646(58.3)	36(65.5)	449(54.8)	30(30.6)	131(97.0)
Female	462(41.7)	19(34.5)	371(45.2)	68(69.4)	4(3.0)
**Incontinence pattern after age 5 years**^**a**^					
Day time	422(53.9)	16(29.0)	329(40.1)	40(40.8)	38(28.1)
Night-time	460(58.8)	18(32.7)	353(43.0)	45(45.9)	43(31.9)
Both	490(62.6)	20(36.3)	374(45.6)	49(50.0)	49(36.3)
**Maximum cystometric capacity (% of EBC)**					
Small (< 65%)	325(29.7)	3(5.5)	275(33.5)	30(30.6)	17(12.6)
Normal (65- 150%)	680(62.3)	52(94.5)	486(59.2)	47(48.0)	95(70.4)
Large (> 150%)	87(8.0)	-	53(6.5)	13(13.2)	21(15.6)
**Low bladder compliance**	492(44.4)	-	420(51.2)	20(20.4)	52(38.5)
**Detrusor activity**					
Overactive	163(14.8)	-	87(10.6)	50(51.0)	26(19.3)
Underactive	50(4.5)	-	26(3.2)	20(20.4)	4(3.0)
**VUR**					
High	41(3.7)	-	21(2.6)	5(5.1)	15(11.1)
Low	13(1.2)	-	8(1.0)	4(4.1)	1(0.7)

*n* = 782, *EBC* = [age (years) × 30 + 30 (expressed in ml)] for those > 2 years old and [ 7 × weight (kg) expressed in ml)] for those < 2 years old

### High risk features for upper tract damage

**Fig. 4:**
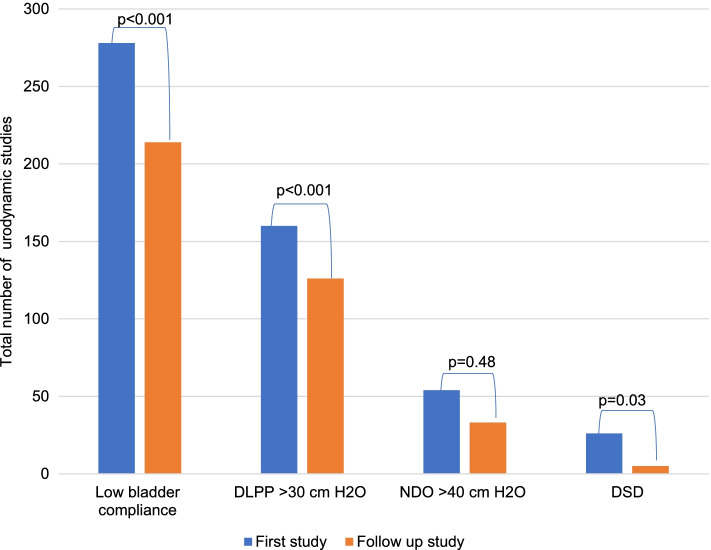
Comparison of bladder dynamics (high-risk features for upper tract damage) between the first and follow-up study

With the exception of neurogenic detrusor overactivity (*p* = 0.48), there was statistically significant improvement in low bladder compliance (*p* < 0.001), detrusor leak point pressure > 30 cm H_2_O (*p* < 0.001) and detrusor sphincter dyssynergia (*p* = 0.03) between the first study and the study following therapeutic intervention (Fig. 4).

## Discussion

This study is a description of a 15-year experience with 1108 invasive urodynamic studies performed at Red Cross War Memorial Children’s Hospital between September 2005 and September 2020. The most important findings of this study are the following: (1) the increasing trends in the use of UDS from 2015; (2) the wide range of indications for urodynamic testing; (3) presence of high risk urodynamic features at first study despite having previously been evaluated using the conventional modalities and receiving multiple bladder treatments; (4) a statistically significant improvement between first time study and follow-up study (post interventional) using low bladder compliance, detrusor sphincter dyssynergia (DSD) and detrusor leak point pressure (DLLP) > 30 cm H_2_O.

In the present study, the majority (58.3%) of UDS were performed on male patients with a median age at first study of 7.0 years (range 4.0–11.0 years). A similar sex difference was also reported by Hoebeke et al. and Swithinbank et al. [11, 14]. The observed male predominance can be explained by the fact that LUTDs such as PUV, which accounted for 13.8% of all studies, only occur in male patients. Studies with a female predominance were mainly focusing on the use of UDS in children with NNLUTD such has urge syndrome, dysfunctional voiding etc. [8, 9, 16].

The 15-year results showed increasing trends in the use of UDS. The observed upward trend from 2015 can be explained by the increase of knowledge surrounding the advantages of using the urodynamic studies when evaluating children with LUTD. Previous research corroborates the benefits of utilizing UDS for the diagnosis and management of children with neurogenic lower urinary tract dysfunction [5, 17–20]. When evaluating 51 patients with closed spina bifida, Johnston et al. demonstrated that clinical neurological assessment, history of voiding habit and renal tract ultrasonography were not reliable indicators of bladder dysfunction compared to VUDS [5]. Tarcan et al. reported that newborns with myelodysplasia and normal bladder function on urodynamics still require follow-up urodynamic testing [17]. Several guidelines including the ICCS, European Association of Urology and the European Society for Paediatric Urology (EAU/ESPU) recommend urodynamic testing for all children with suspected neurogenic lower urinary tract dysfunction [4, 6].

Because of this compelling data on UDS in children with NLUTD, it is not surprising that the majority (62.1%) of urodynamic testing in this study were of children with spinal abnormalities. There may be a preferential referral from the multidisciplinary Spinal Defect Clinic at RCWMCH as the Urologists are the primary discipline at these clinics. A total of 37.7% studies performed were requested by the Urology department. There also seem to be a fair number of referrals coming from other departments, Spinal defect clinic 34.4%, Nephrology 20.8% and other (Oncology, Neurology, Neurosurgery, other hospitals) 7.0%. This finding suggests that the value of UDS has received heightened awareness even in specialties that have historically not involved it. Another notable finding is the small number 49 (4.4%) of patients referred with enuresis as an indication. This might be due to the fact that the centre currently has a tendency of undertaking uroflowmetry first for all functional LUTD and reserving invasive studies for patients not responding to treatment.

The justification for an invasive urodynamic study request is normally based on the assumption that the outcome is likely to affect treatment, when treatment does not lead to its intended outcome or when surgical interventions are planned [7, 10, 12, 21]. The benefits of performing a UDS should also outweigh the risks. The indications for the UDS studies performed are listed in Table 1. The most common reason for UDS were to discern treatment effects (medical 36.5% and surgical 8.5%). The study revealed that at first urodynamic study, 59.1% of children were already receiving more than one type of bladder management. It is evident from this finding that LUTD can be difficult to treat and often requires more than one type of bladder treatment. This observation also raises a concern of delayed referral for some patients. The ICCS advocates a baseline UDS before the age of one year for all patients with spinal dysraphism, even before symptoms start [4]. Early intervention is necessary to decrease significant complications such as end stage kidney disease in these cases. To improve early referral, there should be continued medical education on UDS and quality improvement projects. CIC appears to be the most commonly used method of treatment. This reflects the hospital’s institutional practice of CIC initiation in spinal dysraphism patients at an early age. The most frequently prescribed drug for bladder management was Oxybutynin (305 of 727), compared to Solifenacin succinate (9 of 727) and Mirabegron not being used in any patients in our study. Drug availability and cost might have influenced the choice of medication prescribed. Both Mirabegron and Solifenacin succinate were not available for use in our hospital during the study period as they are not on the drug formulary mainly due to their high cost. However, patients under medical aid cover were able to purchase the medications from private pharmacies.

The remaining UDS were ordered for recurrent UTI (18.6%), VUR (11.2%), baseline study (10.5%), recurrent UTIs associated with VUR (9.9%), pre- and post-transplant (7.0%), pre- surgical intervention (5.7%) and anorectal malformation (4.9%). The clinical significance of invasive UDS in children with NNLUTD still remain the source of controversy in literature [7, 9, 10]. Soygür et al. retrospectively evaluated the role of VUD in the diagnosis and management of voiding dysfunction and found that VUDS did not generally change approach to the patient [7]. In a multicentre controlled trial in children with urge syndrome and dysfunctional, Bael et al. recommends reserving VUD for those who have failed initial treatment [9]. Glazier et al. demonstrated abnormal VUDS in 28 out of 38 children with recurrent UTIs associated with voiding dysfunction [10]. In their study only 5 out of 38 had abnormal VCUG and KUB. From their study they strongly recommend VUDS to be considered in children with recurrent UTIs and a history of voiding dysfunction [10]. To address the current controversies in the additional value of UDS in children with NNLUTD, ICCS advocates for urodynamic testing in children with NNLUTD only if it will guide treatment plans and procedures [12].

There is controversy surrounding the method of bladder catheterization when performing a urodynamic examination. The concern regarding transurethral catheters is that it can affect urethral function and increase leak point pressure (LPP). However, several studies have demonstrated that the use of catheterization does not alter urethral function [22–24]. The ICCS Standardization Report on Urodynamic Studies of the Lower Urinary Tract in Children recommends the use of either methods; however, risks should be weighed against benefit when the suprapubic route is used. In this study almost all (97.5%) urodynamic studies were performed with either a 6F or 7F double lumen transurethral catheters. Voiding phase is often assessed by performing a uroflow first and removal of the catheter after an invasive study, however this study did not determine the number of UDS where the voiding phase was assessed this way. The main reason for those performed using either Mitrofanoff (1.1%) or suprapubic catheter (1.4%) was because the patients were already using the technique for bladder emptying. The use of suprapubic catheterization is not a feasible option in a resource limited setting as it requires hospital admission with theatre time and monitoring space.

Although UDS usage has increased, concerns have been raised that the use of invasive urodynamic testing is not always justified in some patients. Only 5.0% of studies were reported as normal and this finding may reflect the appropriate use of UDS in children. This is comparable to Hoebeke et al. and Johnston et al. who reported 6% and 8% of normal studies respectively [5, 11]. Glazier et al. reported much higher incidence of normal studies when evaluating the utility of VUD in 42 children with UTI and voiding dysfunction [10]. Even though a significant number (430 of 727) of children were receiving more than on type of bladder treatment at first study, the study revealed low bladder compliance (278 of 727), DLPP > 30 cm H_2_O (160 of 727), neurogenic detrusor overactivity (54 of 727) and DSD (26 of 727). This reflects the difficulties in treating some of these patients and that UDS is often warranted to facilitate more specific diagnoses and guide treatment. The higher incidence of low bladder compliance (420 of 820), in those with NLUTD represents the importance of UDS in all patient with suspected NLUTD to identify those at risk for problems. ICCS advocates early urodynamic profiling in these patients and follow-up studies to allow early intervention and decrease significant complications. In this study the incidence of VUR could only be determined after introduction of VUDS, from year 2017. VUR was detected in 54 out of 168 (32%) VUDS. This study did not differentiate whether VUR was primary or secondary.

The clinical significance of performing a urodynamic testing has been demonstrated in this study. The overall goal of treatment of children with LUTDs is to preserve upper tracts and ameliorate or delay progression to ESKD especially in a setting where dialysis and transplantation may not be easily available. From the literature, factors associated with upper tract damage are low bladder compliance, DSD, neurogenic detrusor overactivity (NDO) and DLPP > 40 cm H_2_O. With the exception of NDO (*p* = 0.48), there was significant statistical improvement between the first study and follow-up study using low bladder compliance (*p* < 0.001), DLPP > 30 cm H_2_O (*p* < 0.001) and DSD (*p* = 0.03). Based on this finding, the use of urodynamic examination in children with LUTDs has the potential to lower the incidence rate of renal replacement therapy.

The strength of this study is that it represents the largest number of invasive urodynamic studies in Sub-Saharan region. Another strength of this study is that it included a wide array of clinical indications and broader range of diagnosis. This study also included a diverse set of referring specialities. Nevertheless, this study is not without limitations. This was a single centre study but with large numbers. Other limitations relate to its retrospective design. Some UDS were excluded because of missing data and some studies may have been missed. Also, the incidence of DSD and VUR reported in this study may have been underestimated as they were periods where the centre was unable to perform electromyography and the use of VUDS started after year 2017.

## Conclusions

In conclusion, this study has demonstrated an increased interest in the use of UDS in children. It has highlighted the difficulties in the management of children with LUTDs which may necessitate that multiple follow-up studies be carried to monitor response. The recommendation of early and frequent follow-up UDS for children with spinal dysraphism recommended by ICCS may not be feasible in a resource limited country. For Africa this may mean early start on CIC, prioritizing patients based on their risk for upper tract damage and effective application of bladder and stool diaries, KUB (pre- and post-void residual volume) ultrasonography and VCUG.

## Supplementary Information

**Additional file 1.** 

## Data Availability

This study includes all relevant data. However, on reasonable request, the Corresponding Author will provide additional data.

## Abbreviations

CIC: Clean Intermittent Catheterisation
DSD: Detrusor Sphincter Dyssynergia
EAU: European Association of Urology
EBC: Expected Bladder Capacity
EFP: End-Fill Detrusor Pressure
ESPU: European Society for Paediatric Urology
ESKD: End Stage Kidney Disease
ICCS: International Children’s Continence Society
IQR: Interquartile Range
KUB: Kidney-Ureter-Bladder
DLPP: Detrusor Leak Point Pressure
LUTD: Lower Urinary Tract Dysfunction
NDO: Neurogenic Detrusor Overactivity
NLUTD: Neurogenic Lower Urinary Tract Dysfunction
NNLUTD: Non-Neurogenic Lower Urinary Tract Dysfunction
PUV: Posterior Urethral Valves
RCWMCH: Red Cross War Memorial Children’s Hospital
SPSS: Statistical Package for Social Sciences
TUC: Transurethral Catheterisation
UDS: Urodynamic Study
UTI: Urinary Tract Infection
VCUG: Voiding Cystourethrogram
VUDS: Video Urodynamic Study
VUR: Vesicourethral Reflux
