# Supplementary material for: A 15-year retrospective review of urodynamic studies in children at Red Cross War Memorial Children’s Hospital, Cape town, South Africa
Source: BMC Pediatr. 2022 Jul 8;22:401. doi: 10.1186/s12887-022-03462-4 (PMC9263046; doi:10.1186/s12887-022-03462-4)
Supplement: Supplementary file 1 — Additional file 1. [file 12887_2022_3462_MOESM1_ESM.docx]

P O Box 2796

Gaborone

Botswana

28 June 2022

To the Editor

BMC Paediatrics

Dear Sir/ Madam

**RESPONSE TO THE REVIEWER’S COMMENTS**

**MANUSCRIPT BPED-D-22-00059R3**

**“A 15-year retrospective review of urodynamic studies in children at Red Cross War Memorial Children’s Hospital in Cape town, South Africa”**

We thank the reviewers for their valuable comments and recommendations. The constructive suggestions from the reviewers have greatly enhanced the quality of this manuscript.

We have addressed the reviewers’ comments and the responses are below. We have also modified the manuscript as noted in our responses

Thank you for your consideration of this manuscript.

Sincerely,

Thembisile Dintle Mosalakatane

[dintletn@yahoo.com](mailto:dintletn@yahoo.com)

(corresponding author)

**RESPONSE TO THE REVIEWER’S COMMENTS**

**MANUSCRIPT BPED-D-22-00059R3**

**“A 15-year retrospective review of urodynamic studies in children at Red Cross War Memorial Children’s Hospital in Cape town, South Africa”**

***Before we can continue we would ask that you move the information regarding funding from the "Acknowledgements" to the "Funding" section. In Funding, please also state whether or not the funding body played any roles in the design of the study and collection, analysis, and interpretation of data and in writing the manuscript.***

We thank the Reviewer for this suggestion. The following information was moved from “Acknowledgements” to the “Funding” section *(page 23, funding section line 1):*

“This study was done as part of TDM’s Master of Philosophy in Paediatric Nephrology and the authors would like to acknowledge the International Society of Nephrology (ISN) and International Paediatric Nephrology Association (IPNA) for funding her training. “This work has been made possible through her ISN-IPNA funded Fellowship”.

The funding section was modified as shown below *(page 23, funding section):*

“No funding was obtained for this study. This study was done as part of TDM’s Master of Philosophy in Paediatric Nephrology and the authors would like to acknowledge the International Society of Nephrology (ISN) and International Paediatric Nephrology Association (IPNA) for funding her training. “This work has been made possible through her ISN-IPNA funded Fellowship”. The funding was solely for the paediatric nephrology training and not for the design of the study, data collection, analysis and interpretation and in the writing of the manuscript.”
